# Discovery of Novel HIV Protease Inhibitors Using Modern Computational Techniques

**DOI:** 10.3390/ijms232012149

**Published:** 2022-10-12

**Authors:** Sunday N. Okafor, Pavimol Angsantikul, Hashim Ahmed

**Affiliations:** 1 Center for Biomedical Research, Population Council, New York, NY 10065, USA; 2 Department of Pharmaceutical and Medicinal Chemistry, University of Nigeria, Nsukka 41001, Nigeria

**Keywords:** HIV protease, ADMET, pharmacophore, molecular docking, HIV protease inhibitors

## Abstract

The human immunodeficiency virus type 1 (HIV-1) has continued to be a global concern. With the new HIV incidence, the emergence of multi-drug resistance and the untoward side effects of currently used anti-HIV drugs, there is an urgent need to discover more efficient anti-HIV drugs. Modern computational tools have played vital roles in facilitating the drug discovery process. This research focuses on a pharmacophore-based similarity search to screen 111,566,735 unique compounds in the PubChem database to discover novel HIV-1 protease inhibitors (PIs). We used an in silico approach involving a 3D-similarity search, physicochemical and ADMET evaluations, HIV protease-inhibitor prediction (IC_50_/percent inhibition), rigid receptor–molecular docking studies, binding free energy calculations and molecular dynamics (MD) simulations. The 10 FDA-approved HIV PIs (saquinavir, lopinavir, ritonavir, amprenavir, fosamprenavir, atazanavir, nelfinavir, darunavir, tipranavir and indinavir) were used as reference. The in silico analysis revealed that fourteen out of the twenty-eight selected optimized hit molecules were within the acceptable range of all the parameters investigated. The hit molecules demonstrated significant binding affinity to the HIV protease (PR) when compared to the reference drugs. The important amino acid residues involved in hydrogen bonding and п-п stacked interactions include ASP25, GLY27, ASP29, ASP30 and ILE50. These interactions help to stabilize the optimized hit molecules in the active binding site of the HIV-1 PR (PDB ID: 2Q5K). HPS/002 and HPS/004 have been found to be most promising in terms of IC_50_/percent inhibition (90.15%) of HIV-1 PR, in addition to their drug metabolism and safety profile. These hit candidates should be investigated further as possible HIV-1 PIs with improved efficacy and low toxicity through in vitro experiments and clinical trial investigations.

## 1. Introduction

HIV protease (PR) is one of the three enzymes essential in the life cycle of the human immunodeficiency virus (HIV) survival and replication. At some point in the life cycle of HIV, immature viral particles are produced. These budded immature viral particles that contain catalytically inactive protease cannot undergo maturation to an infective form [1]. The role of PR is basically to catalyze the hydrolysis of Gag and Gag-Pol polyproteins thereby generating mature infectious virions [2]. HIV protease inhibitors work by antagonizing the process that leads to the generation of mature infectious virions. When there is a chemical inhibition of this enzyme or there is a mutation in the catalytic residues of the PR, there is a production of immature noninfectious viral particles which lacks the capacity for viral infectivity [1,3,4,5]. Therefore, HIV PR has played a key role as an attractive target for the design of drugs against acquired immunodeficiency syndrome (AIDS) [6]. HIV protease inhibitors are a class of anti-HIV drugs that inhibit the hydrolysis of Gag and Gag-Pol polyproteins, which ultimately prevents the production of mature infectious virions [7]. Currently, the FDA has approved ten protease inhibitors for clinical use. They are saquinavir, indinavir, ritonavir, nelfinavir, amprenavir, fosamprenavir, lopinavir, atazanavir, tipranavir and darunavir.

Over the decades, computer-aided drug design (CADD) has brought tremendous breakthroughs in the field of drug discovery [8,9]. Basically, CADD is used to catalyze and rationalize the drug design process while reducing costs and time [10,11]. DiMasi et al. [12] and Song et al. [13] have reported that an average of 10–15 years and an average of USD 800 million will be required to bring a new drug into the market. Paul and co-workers, in 2010, approximated the cost of drug discovery to USD 1.8 billion [14]. CADD explores computational techniques to study the drug–receptor interactions and to determine the binding potential and affinity of a given molecule to a drug target [15]. Different chemical interactions such as hydrophobic, electrostatic and hydrogen bonding are seen in the binding of ligands to the receptor. It has been applied in the earliest stage in drug discovery to identify hit compounds by high throughput screening (HTS) [11]. Virtual screening focuses on filtering libraries of compounds using in silico methods to prioritize those compounds that are most likely going to be active against a selected target. Subsequently, these hits and lead molecules are tested with a suitable activity assay for potency and ADMET properties. Notable among the modern techniques in CADD is pharmacophore modeling.

Pharmacophore is a molecular framework that carries the essential features (phoros) responsible for a drug’s biological activity (pharmacon) [16]. Similarly, the International Union of Pure and Applied Chemistry (IUPAC) has defined pharmacophore as “the ensemble of steric and electronic features that is necessary to ensure the optimal supra-molecular interactions with a specific biological target structure and to trigger (or to block) its biological response” [17,18]. There are so many applications of pharmacophore modeling in the drug discovery process ranging from virtual screening to target identification, scaffold hopping, ligand profiling, lead optimization and de novo drug design. Therefore, this method is a widely used tool in CADD and chemoinformatics fields [19].

**Figure 1:**
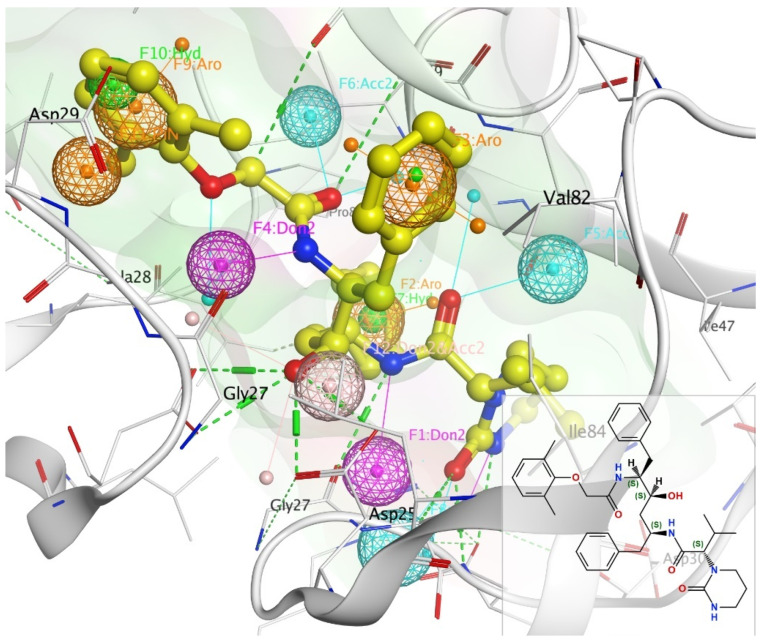
Pharmacophore annotation of lopinavir bound to 2Q5K using unified scheme. **Key**: H-bond donor—Don (pink), H-bond acceptor—Acc (cyan), hydrophobic atom—HydA (green), projected donor—Don2 (pink), projected acceptor—Acc2 (cyan), aromatic—Aro (orange).

Pharmacophoric modeling is based on the theory that two or more compounds that have common chemical functionalities, and maintain a similar spatial arrangement, will lead to similar biological activity on the same target [20]. The key important pharmacophoric feature types are hydrogen bond donors (HBDs); hydrogen bond acceptors (HBAs); hydrophobic areas (H); positively and negatively ionizable groups (PI/NI); aromatic groups (AR); and metal coordinating areas (Figure 1). Additional size restrictions in the form of shape or exclusion volumes (XVOL)—forbidden areas—can be added to represent the size and shape of the binding pocket [21]. Pharmacophore modeling can be structure-based or ligand-based. In this study, we have used ligand-based pharmacophore modeling to screen the PubChem database for compounds with bioactivity against HIV-1 protease.

## 2. Results and Discussion

### 2.1. Pharmacophore Annotation and Similarity Search

The basic annotation features of lopinavir bound to HIV-1 protease were created as shown in Figure 1: Atom (H-bond donor—Don, H-bond acceptor—Acc, hydrophobic atom—HydA); Projected (projected donor—Don2, projected acceptor—Acc2, ring normal—PiN), and Centroid (aromatic—Aro, pi-ring—PiR, hydrophobic—Hyd). Pharmacophore annotations for other reference drugs are shown in Figure S2.

**Figure 2:**
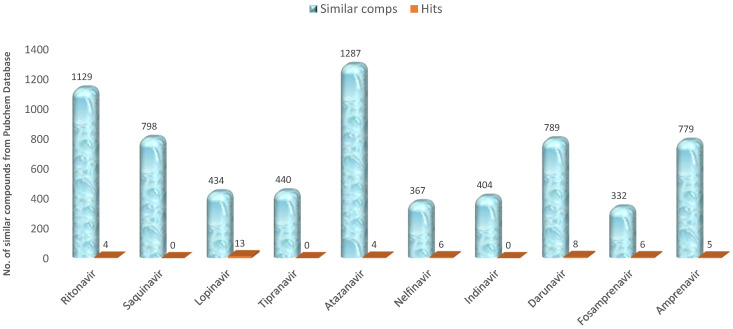
Similarity search outcome using different HIV I protease inhibitors as reference drugs.

The result of the pharmacophoric similarity search is summarized in Figure 2. Preliminary filtration was performed, where compounds that had more than one Lipinski violation were filtered off. The search result returned a total of 6759 similar compounds from different HIV protease inhibitors used as reference (Figure 2). At the end of further screening and optimization of these compounds, we obtained 46 hits (Figure 2). We noted that there were no hits from saquinavir, tipranavir and indinavir based on our screening criteria. The screening criteria used to exclude molecules include violation of more than 2 Lipinski’s parameters, binding affinity less than the reference drug (from the docking scores), low anti-HIV protease inhibition activity, poor ADME properties and potential toxicity. The molecules from saquinavir, tipranavir and indinavir failed in one or more of the above criteria.

### 2.2. Physicochemical Properties Evaluation

Table 1 shows the physicochemical properties of the compounds which are important factors in the determination of the solubility, permeability, and bioavailability of an orally delivered drug. With the exceptions of HPS/013, HPS/014, HPS/015, HPS/016, and HPS/017, we noted that the molecular weights of most of the compounds are greater than 500 Da. This is not so surprising as the molecular weights of all the reference drugs used were more than 500 Da. However, other parameters for drug-likeness according to Lipinski’s rule of five (Ro5) were obeyed, ensuring that the compounds did not violate more than one of Lipinski’s rules. Traditionally, therapeutics have been small molecules that fall within Lipinski’s rule of five (i.e., a molecule with a molecular mass less than 500 Da, no more than 5 hydrogen bond donors, no more than 10 hydrogen bond acceptors, and an octanol–water partition coefficient log P not greater than 5) [22]. Violation of not more than one rule is a good indication of molecules that will have good oral bioavailability. It should be noted that compounds that are substrates for biological transporters do not obey this rule, they can have violations up to 4 [23].

The partition coefficient (logP) is the measure of the lipophilicity of a drug and an indication of its ability to cross the cell membrane. It has served as one of the fundamental principles for drug discovery and design [24]. Maintaining a balance in octanol–water partition coefficient of a molecule is very important. All the hit molecules are within the range of Lipinski’s Ro5. When there is high lipophilicity, there will be a high rapid metabolic turnover of the [25], low solubility and poor absorption of the compound. Furthermore, an increase in lipophilicity increases the probability of binding to hydrophobic protein targets that are not the desired ones, leading to an increase in the potential for toxicity. Log P and other physicochemical properties are of potential importance in the prediction and optimization of drug-likeness of molecules.

Topological polar surface area (TPSA) is a molecular descriptor widely used in the study of drug transport properties such as intestinal absorption [26] and blood–brain barrier (BBB) penetration [27]. In addition, the polar surface area (PSA), which reflects the ligand hydrophilicity, is very vital in protein–ligand interaction [28]. The major application of TPSA in medicinal chemistry has been to evaluate how molecules can cross different membrane barriers [29]. Kelder and colleagues showed drug intestinal permeation predominated by passive diffusion and paracellular route for drugs with TPSA of less than 120 Å [30]. Compounds with a TPSA >140 Å have been identified as poorly absorbed. Most of our hit molecules have TPSA < 140 Å. The percentage solubility calculated from % ABS = 109–0.345 × TPSA [31], ranges from 60.56–72.75%. Both the TPSA and the % ABS showed that most of our hit molecules had good solubility and are well absorbed, a designation of good bioavailability upon oral administration.

### 2.3. Molecular Docking Studies

**Figure 3:**
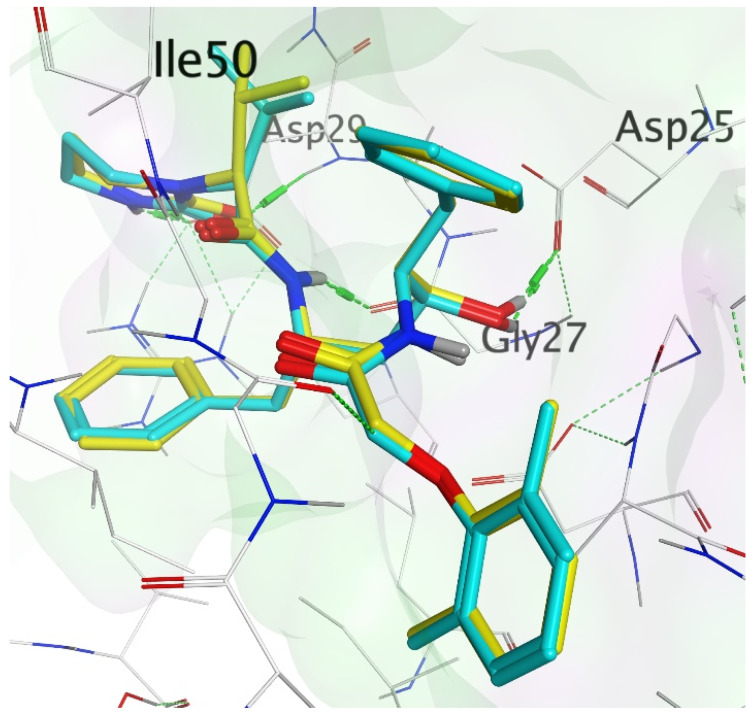
Validation of the docking protocol with HIV-1 protease bound to lopinavir. The docked lopinavir (green) was overlaid on the co-crystallized lopinavir (yellow).

**Figure 4:**
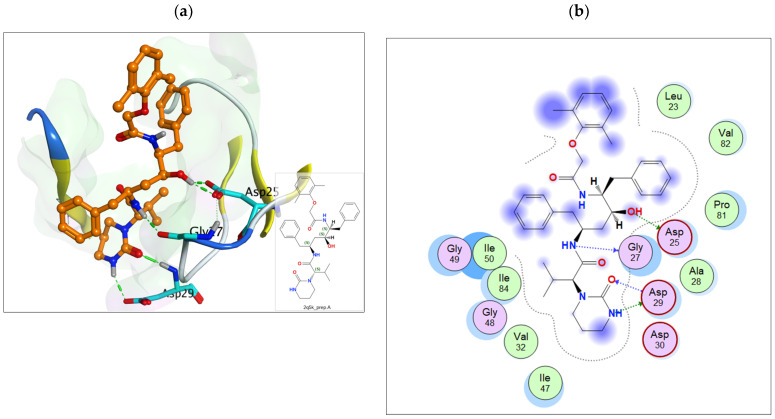
(**a**) The 3D and (**b**) 2D representation of lopinavir binding to the catalytic triad of 2Q5K.

**Figure 5:**
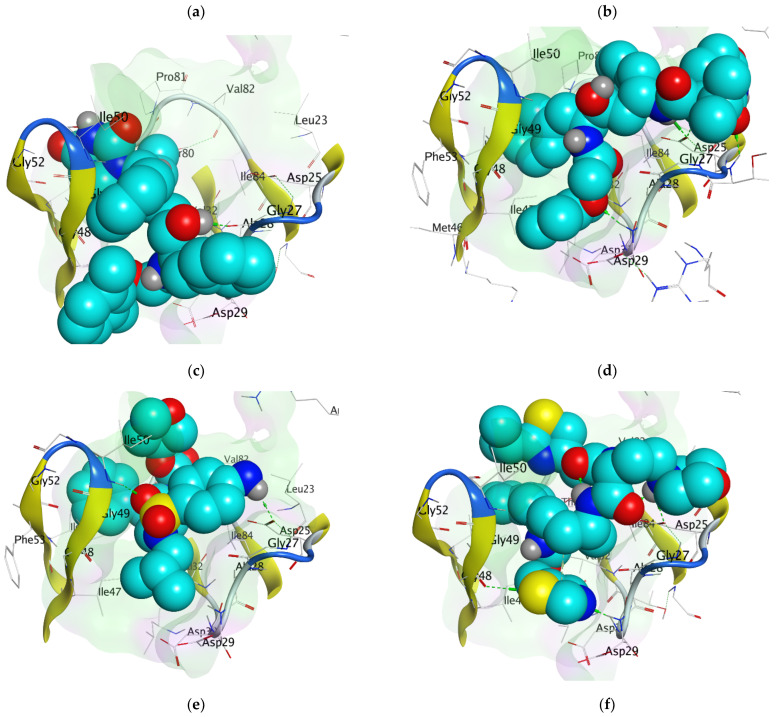

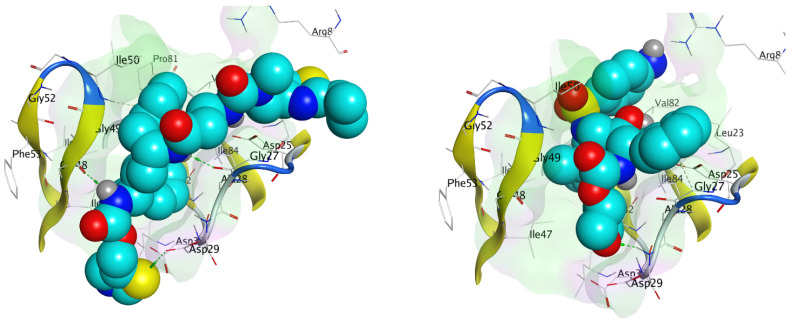
The docking poses of (**a**) HPS/002, (**b**) HPS/004, (**c**) HPS/006, (**d**) HPS/007, (**e**) HPS/008 and (**f**) HPS/009 in the binding cavity of HIV-1 protease.

Molecular docking accurately predicts the experimental interaction mode and ligands affinity within the active binding site of the drug target, making it very significant in the discovery of new drugs [32]. Using the SiteFinder in MOE, the following amino acid residues were found to be part of the active binding site of the 2Q5K: ASP25, GLY27, ALA28, ASP29, ASP30, VAL32, ILE47, GLY48, GLY49, ILE50, GLY52, PHE53, ILE54, THR80, PRO81, VAL82 and ILE84. The docking protocol was further validated by docking the retrieved co-crystallized ligand (lopinavir) into the binding cavity of the 2Q5K-liponivir complex (Figure 3) and measuring the root mean square deviation (RMSD). The RMSD gave 0.9 Å. Usually, RMSD ≤ 2 Å for a pose is considered a docking success. Our docking protocol was, therefore, fully validated. Figure 3 shows the docked ligand (cyan) almost perfectly overlapped the co-crystallized ligand (yellow). Figure S1 of the supplementary material also gives more insight into the validation, where both the docked and the co-crystallized lopinavir shared the same amino acid residues of the catalytic triads (ASP 25, GLY 27 and ASP 29) as shown in Figure 4. Table 2 shows the binding free energy, ΔG (kcal/mol) from the molecular docking calculations using MOE. The ΔG of the hit molecules is lower than the reference drug used. The low ΔG (high negative values) is an indication of the high binding affinity of these molecules with HIV protease. All the hit molecules have higher binding affinity than their respective reference drugs. These docking scores suggest that the hit molecules might have better HIV-1 protease inhibitory activities. HPS/005 and HPS/006 showed the most favorable docking scores of −8.98 and −8.63 kcal/mol, respectively. Due to toxicity concerns of HPS/005 in Table 3 and Table 4, it was not further studied. Hit molecules HPS/002, HPS/004, HPS/006, HPS/007, HPS/008 and HPS/009 were selected for further studies in order to gain more insight into the nature of the chemical interactions and conformations of docked compounds. The criteria for their selection include high binding affinity when compared to the reference, significant anti-HIV activity (low IC_50_ and high % inhibition), good ADME properties and low toxicity potential. The docking poses were visualized, and the detailed interactions were recorded as shown in Figure 5 and Table 3, respectively.

Molecular docking simulation clearly demonstrated significant chemical interactions of the atoms of the ligands and the amino acid residues of the HIV protease (Table 3). The amino acid residues of the HIV-1 protease that interacted with the atoms of the optimized hit compounds (Table 3) are all part of the amino acid residues in the active binding sites. This further elucidates the validity of the docking protocols. All the hit molecules formed prominent hydrogen bonds with at least two catalytic residues (ASP 25, GLY 27 and ASP 29) on the floor of the active site. The carbonyl and the hydroxy group of HPS/002, through hydrogen bond interactions, combined with the O GLY 27 and N ASP 29, respectively. Other important chemical interactions include the pi-H bonding of the two 6-membered aromatic rings with the CG1 of ILE 50 and the CA of GLY 27, respectively. HPS/004 has similar interactions to those exhibited by HPS/002. HSP/002 and HPS/004 have more effective binding interactions with the HIV-1 PR than lopinavir. In addition to using the amino acid residues of the catalytic triad, they also interact effectively with other amino acid residues. These interactions help to stabilize them more in the active binding site of the receptor. The two 5-membered thiazole rings in HPS/007 interacted with NH1 ARG 8, CA GY 49 and CB PRO 81 through pi-H bonding. Furthermore, the S-2 and O-7 atoms of the HPS/007 inhibitor form a hydrogen bond with the CG1 of ILE 50 and CA of GLY 49, respectively. The sulfur atom (S-1) in the thiazole ring of HPS/006 formed two strong H-bonds with the OG1 THR 80 and CD1 ILE 54 through H-donor and H-acceptor interactions, respectively. Furthermore, the N-10 atom of the HPS/006 compound interacted with the O GLY 27, forming a strong H-bond. Other amino acid residues that played a vital role in the chemical interactions of HPS/006 and the HIV protease are ASP 25, by interacting with C-29, and GLY 49, by interacting with O-4. Likewise, the S-2 in the thiazole ring of HPS/008 interacted with the carboxyl group of the protease active site residues, ASP 29 and ASP 30 through hydrogen bonding. There were prominent hydrogen bond interactions between the 6-membered rings of HPS/008 and the CD1 of ILE 47, and amino nitrogen of ILE 50 through pi-H bonding. Other amino acid residues that played vital roles in the interactions are: GLY 27, GLY 48 and LYS 45. It was observed that HPS/007 has the highest binding affinity (−8.57 kcal/mol). In addition to the chemical interactions with the residues in the catalytic active site, the two thiazole rings contributed significantly to this high binding affinity of HPS/007 with the HIV protease. Again, the 6-membered aromatic rings, which are common in all the hit molecules played a key role in the chemical interactions, which resulted in an improved binding affinity of these molecules to the HIV protease. Figure 5 illustrates the CPK representation of the binding poses of (a) HPS/002 (b) HPS/004 (c) HPS/006 (d) HPS/007 (e) HPS/008 and (f) HPS/027 in the binding cavity of HIV-1 protease.

### 2.4. Anti-HIV Prediction Analysis

The results of HIV protease inhibitory activity—IC_50_ (µM) and % inhibition of the selected hits from different reference drugs using HIVprotI are shown in Table 2. The fourteen selected hit molecules showed significant activity against HIV-1 protease with IC_50_ range of −2.52–48.90 µM and 52.71–90.13% inhibition. Generally, each selected hit molecule has a better IC_50_ and/or % inhibition when compared to its reference drug. HPS/002 has a comparable % inhibition of 90.13% to its reference drug—lopinavir (90.12%). However, it showed lower and better IC_50_ of 48.90 µM than lopinavir (203.69 µM). A low IC_50_ value implies that the drug candidate is potent at a low concentration and ultimately may show lower systemic toxicity when administered to the patient.

### 2.5. ADMET Analysis

The pharmacokinetic and pharmacodynamic profiles are usually used to assess the safety and efficacy of a drug during the drug development process. Despite how promising a drug candidate may be, the ADMET drug properties determine the extent to which it can be useful as a drug. We have extensively evaluated the ADMET properties that can limit the use of drug candidates. Various ADMET properties of the optimized hit molecules were evaluated. The information can be used to predict various pharmacokinetic phenomena of these compounds, which ultimately guide the further development of new drug compounds [33]. A compound that does not meet certain ADME standards may result in poor bioavailability. In like manner, compounds which are very toxic cannot be useful as drug candidates as the risks will outweigh the potential benefits of such compounds.

#### 2.5.1. Absorption and Distribution Evaluations

Water solubility is an essential aspect of a drug’s pharmacological reaction following oral delivery. Drugs with strong water solubility will have good absorption and bioavailability qualities. Drug absorption and bioavailability can boost plasma drug concentrations at the target location, allowing it to fulfill therapeutic actions [34]. The compounds showed good water solubility (Table 4). The absorption parameters shown in Table 4 indicate that the compounds could be absorbed from the intestinal tract upon oral administration. All the hit molecules have high GI absorption (poorly absorbed <30%), denoting an increase in permeability. High Caco-2 cell permeability is associated with a permeability value > 0.9 [35] The hit molecules and all the FDA-reference drugs except Lopinavir did not comply with threshold values. Table 4 also shows the distribution parameters analysis. For a given compound, a logBB > 0.3 is considered to readily cross the blood–brain barrier while molecules with logBB < −1 are poorly distributed to the brain. Similarly, compounds with a logPS > −2 are considered to penetrate the central nervous system (CNS) while those with logPS < −3 are considered unable to penetrate the CNS. From Table 4, all the hit molecules, including the reference drugs are not readily able to cross the blood–brain barrier (BBB) or penetrate the central nervous system (CNS). The only exception to this is lopinavir, which can penetrate the CNS but was not readily able to cross the BBB. The volume of distribution, VDss is the theoretical volume that the total dose of a drug would need to be uniformly distributed to give the same concentration as in blood plasma. The higher VD is the more of a drug is distributed in the tissue rather than plasma. VDss is considered low if below 0.71 L/kg (log VDss < −0.15) and high if above 2.81 L/kg (log VDSS > 0.45) [35]. While some of the hit molecules have a high volume of distribution at a steady state (VDss), others have a low to moderate VDss.

#### 2.5.2. Metabolism and Excretion

Table 5 shows the metabolism and excretion parameters analysis. CYP1A2, CYP2C19, CYP2C9, CYP2D6, CYP2E1 and CYP3A4 play key roles in drug metabolism. The results suggest that except for lopinavir, HPS/015, HPS/019, HPS/024 and HPS/028, all the other hit molecules and reference drugs inhibit the CYP3A4 sub-enzymes of cytochrome P450 (CYP). CYP3A4 is responsible for metabolizing ∼50% of all drugs by itself [36]. All the hit molecules and the reference drugs do not inhibit CYP2D6 except HPS/017, HPS/028 and nelfinavir. Most of the compounds and drugs evaluated are not CYP2C9 inhibitors while others inhibit it. It has been demonstrated that CYP2C9 can metabolize some marketed drugs [36].

All except HPS/002 are organic cation transporter 2 (OCT2) non-substrates. OCT2 is a renal uptake transporter that plays a critical role in the disposal and renal clearance of drugs and endogenous compounds [36]. OCT2 substrates can adversely interact with co-administered OCT2 inhibitors. This evaluation has provided insightful information on drug clearance and potential contraindications of OCT2 transporter drug candidates.

#### 2.5.3. Toxicity Analysis

The LD_50_ (mg/kg body weight) is the median lethal dose at which 50% of test subjects die upon exposure to a compound. The Globally Harmonized System of classification of labeling of chemicals (GHS) has categorized LD_50_—mg/kg into the following classes: Class I: fatal if swallowed (LD_50_ ≤ 5); Class II: fatal if swallowed (5 < LD_50_ ≤ 50); Class III: toxic if swallowed (50 < LD_50_ ≤ 300); Class IV: harmful if swallowed (300 < LD_50_ ≤ 2000); Class V: may be harmful if swallowed (2000 < LD_50_ ≤ 5000) and Class VI: non-toxic (LD_50_ > 5000). None of the hits is fatal when swallowed as shown in Table 6. Some are toxic when swallowed while others may be harmful when swallowed. This is an affirmation that drugs are poisons and not food that is non-toxic when swallowed (Class VI). This harmful nature may have to do with some side effects that may be associated with drugs when taken. In Table 6, almost all the hits are found to be inactive, with a high probability, to hepatotoxicity, carcinogenicity, immunotoxicity, cytotoxicity, aromatase, estrogen receptor-α, androgen receptor and peroxisome proliferator-activated receptor gamma. This finding gives more credence to our hit compounds as potential HIV protease inhibitors. The androgen receptor (AR) is the main biomolecular target involved in the development and progression of hormone-dependent prostate cancer [37]. In silico evaluation of small-molecule binding at the AR is very important for the screening of potential androgen-disrupting chemicals [38] causing endocrine disorders [39,40]. Additionally, disruption of the androgen system is associated with decreased sperm count, increased infertility [41] and diabetes mellitus [42]. We have predicted from this study that ritonavir, atazanavir and darunavir have hepatotoxicity potentials (Table 6).

The AMES (the bacterial reverse mutation test) toxicity profile (Table 7) indicates that the hit molecules do not have potential mutagenic tendencies. It is only PC_HPS/017 that tests positive and therefore may act as a carcinogen since HIV-1 is often linked to mutation. The maximum recommended tolerated dose (MRTD) provides an estimate of the toxic dose threshold of chemicals in humans. For a given compound, an MRTD of ≤20.477 log(mg/kg/day) is considered low, and higher >0.477 log log(mg/kg/day). All the hit molecules showed values less than the maximum human-tolerated dose (0.477 log mg/kg/day), indicating no possible dose-related toxicity. None of the hit molecules, including the reference drugs, are considered a likely inhibitor of hERGI. HPS/002, HPS/004, HPS/007, HPS/023, HPS/027 and some other hit molecules are considered possible hERGII inhibitors while HPS/015 and some other hits will potentially not inhibit hERGII. Human ether-a-go-go-related gene (HERG) potassium channels are expressed in multiple tissues including the heart and adenocarcinomas [43]. The pharmacological reduction of HERG currents may cause acquired long QT syndrome and life-threatening “torsade de pointes” arrhythmias [44]. HERG expression in tumor cells accelerates cell proliferation [45], and inhibition of HERG currents has been shown to reduce cell proliferation [46]. The hepatotoxicity result shows that the hit molecules and all the FDA-approved drugs used as reference could be related to at least one physiological or pathological event, which could be associated with disruption of normal liver function. *T. pyriformis* is a protozoa bacterium with its toxicity measured in log μg/L often used as a toxic endpoint. If the value is <−0.5 log μg/L for any compound, it is considered to be toxic [47]. None of the hit molecules and the reference drugs showed toxicity against *T. pyriformis*. Similarly, Minnow toxicity was assessed. It is an indication of the lethal concentration (LC_50_) that represents the concentration of a molecule required to cause the death of 50% of the flathead minnows. A compound with log LC_50_ < −0.3 is regarded as having high acute toxicity [36]. While some hit molecules may be associated with minnow toxicity, others are not. In the light of the foregoing discussions, we observe that the majority of the hit molecules’ predicted toxicities (Table 6 and Table 7) maintain a relatively lower acute toxicity risk compared to reference drugs.

## 3. Materials and Methods

### 3.1. Database

The database for this study is PubChem (https://pubchem.ncbi.nlm.nih.gov/; accessed on 30 March 2022). It is an open chemistry database maintained by the National Institutes for Health (NIH). It was launched in 2004 and has remained a veritable chemical information resource for scientists involved in drug discovery research. PubChem is the world’s largest freely accessible database containing approximately 111 M compounds, 281 M substances, 295 M bioactivities, 34 M pieces of literature, and 42 M patents.

### 3.2. Physicochemical Evaluation for Drug-Likeness

The Molecular Descriptors Algorithm in MOE was used to evaluate the physicochemical properties of the compounds. These properties include molecular weight (MW), log octanol/water partition coefficient (log P), number of H-bond donor atoms (a_don), number of H-bond acceptors (a_acc), topological polar surface area (TPSA), number of rotatable bonds (b_rotN). We applied Lipinski’s rule of five to evaluate the drug-likeness. The result is shown in Table 1.

### 3.3. ADMET Predictions

pkCSM was used to evaluate the pharmacokinetic (absorption, distribution, metabolism and excretion), as well as toxicity (ADMET) properties of the screened compounds [36]. pkCSM is a free online webserver for the prediction of small-molecule pharmacokinetic and toxicity properties using graph-based signatures (http://structure.bioc.cam.ac.uk/pkcsm; accessed on 30 March 2022). The pharmacokinetic properties for absorption include water solubility, Caco-2 permeability, intestinal absorption (human), skin permeability, P-glycoprotein substrate, P-glycoprotein I inhibitor and P-glycoprotein II inhibitor; distribution: VDss (human), fraction unbound (human), BBB permeability and CNS permeability; metabolism: CYP2D6 substrate, CYP3A4 substrate, CYP1A2 inhibitor, CYP2C19 inhibitor, CYP2C9 inhibitor, CYP2D6 inhibitor and CYP3A4 inhibitor; excretion: total clearance and renal OCT2 substrate; toxicity: AMES toxicity, maximum tolerated dose (human), hERG I inhibitor, hERG II inhibitor, oral rat acute toxicity (LD50), oral rat chronic toxicity (LOAEL), hepatotoxicity, skin sensitization, *T. Pyriformis* toxicity and minnow toxicity. The results of these evaluations are shown in Table 4, Table 5, Table 6 and Table 7.

We went further to evaluate organ toxicity (hepatotoxicity), toxicity endpoints (carcinogenicity, immunotoxicity, mutagenicity, and cytotoxicity), hormonal-related toxicity (androgen receptor, aromatase, estrogen receptor alpha, and peroxisome proliferator-activated receptor gamma (PPAR-Gamma) using ProTox II as shown in Table 3 [48]. The acute toxicity measured as LD_50_ was equally predicted. The ProTox II can be assessed with this link: http://tox.charite.de/protox_II (accessed on 30 March 2022).

### 3.4. Pharmacophore Generation and Similarity Search

Pharmacophore Query Editor in MOE was used to annotate the 2Q5K-lopinavir complex loaded in MOE as well as rendering the resulting annotations graphically in the MOE window (Figure 1). Annotation is the process of identifying regions of pharmacophoric importance in space around a molecular conformation and associating with those regions of pharmacophore types. The unified annotation scheme was used. In addition to the search mechanism in the PubChem database, this query was also used to search against the database to identify the similar pharmacophore model among them and separate the active compounds from the rest of the database.

### 3.5. Molecular Docking (MOE, 2020) [49]

**Figure 6:**
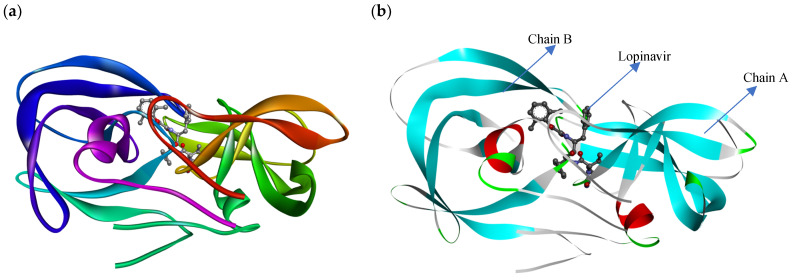
The 3D structure of 2Q5K for molecular modeling: (**a**) publication quality representation, (**b**) flat ribbon representation (all prepared using Discovery Studio Visualization vs21.1.0.20298).

The crystal structure of the wild type HIV-1 protease co-crystallized with lopinavir at a resolution of 1.95 Å (Figure 6) was obtained from the Protein Data Bank (PDB ID: 2Q5K) [50], and loaded into Discovery Studio. The preliminary preparation of the protein was performed in Discovery Studio where the water molecules and heteroatoms were eliminated. The 2Q5K was then loaded into the MOE. The Quickprep algorithm in MOE comprising structure preparation and protonate 3D was used to complete protein preparation. The polar hydrogens were added. A temperature of 300 K, a salt concentration of 0.1 and pH 7 was specified in the implicit solvated environment to carry out the protonation process. Then, the structure was energy-minimized in the MMFF94x force field to an RMS gradient of 0.1 kcalmol^−1^/Å^2^.

The Finder Site in MOE was used to calculate the possible active sites in a receptor from the 3D atomic coordinates of the receptor—2Q5K, which will help to determine potential sites for ligand binding docking calculations. The Site Finder methodology is based upon Alpha Shapes. The generated sites are ranked according to their Propensity for Ligand Binding (PLB) score, which is based on the amino acid composition of the pocket [51].

The dataset from the outputs of the pharmacophore-based similarity search was prepared and loaded into the MOE where they were further prepared and energy-minimized. All the hit molecules were docked as a database into the predicted binding domain, and 30 conformations were generated for each molecule in the 2Q5K binding site. The triangular matcher placement and rigid receptor refinement were employed. Among them, the conformation with the lowest docking score was chosen to study the binding orientations of the ligands, and each complex was assessed and ranked by the London Δ*G* energy scoring function.

### 3.6. Prediction of HIV Activity

In this study, we used HIVprotI to predict the anti-HIV activity of the selected hits. The half maximal inhibitory concentration. IC_50_ (µM) and the percentage inhibition (%values) of these compounds were evaluated in silico. HIVprotI is a web-based algorithm for the prediction and design of protein-specific anti-HIV compounds (http://bioinfo.imtech.res.in/manojk/hivproti; accessed on 30 March 2022). This web tool was developed by experimentally testing the IC_50_ and percentage inhibition activity of various inhibitors datasets against all three HIV proteins (reverse transcriptase RT, protease, PR, and integrase, IN). These inhibitors from the ChEMBL database were further used to develop support vector machine (SVM)-based quantitative structure–activity relationship (QSAR) models using the inhibitor features, descriptors and fingerprints [52].

## 4. Conclusions

Pharmacophore-guided 3D-similarity search, ADMET profiling, molecular docking studies, and in silico evaluation of anti-HIV activity were carried out on the PubChem database containing 111,566,735 compounds to evaluate potential new antiviral agents against HIV-1 protease. The in silico analysis revealed that fourteen (HPS/002, HPS/004, HPS/006. HPS/007, HPS/008, HPS/009, HPS/010, HPS/011, HPS/012, HPS/013, HPS/014, HPS/018, HPS/020 and HPS/024) out of the twenty-eight selected optimized hit molecules were within the acceptable range of all the parameters investigated, such as physicochemical and ADMET parameters, the predicted IC_50_/percent inhibition of HIV PR protein, docking scores and free binding energies.

There are clear indications from the docking results that residues ASP25, GLY27, ASP29, ASP30, and ILE50 are involved in essential hydrogen bonding and п-п stacked interactions stabilized the optimized hit molecules from PubChem in the active binding site of the HIV-1 PR (2Q5K), thereby playing vital roles for the observed anti-HIV activity. The MD simulation showed that the molecules are stable in the active binding site of the HIV-1 protease. Out of the fourteen hit candidates, HPS/002 and HPS/004 have been found to be most promising in terms of IC_50_/percent inhibition of HIV-1 PR and MD stability, in addition to their drug metabolism and safety profiles. We, therefore, propose that these fourteen hit molecules with non-toxic and good bioavailability predicted qualities, with emphasis on HPS/002 and HPS/004, should be investigated further as possible PR inhibitors through wet lab experiments (in vitro/in vivo and pre-clinical) and clinical trial investigations. This is on the premise that computational studies alone are not sufficient by themselves in the drug discovery process.

## Figures and Tables

**Table 1:** The physicochemical properties of the selected hits.

S/N	Comp Code	MW	a_don	a_acc	b_1rotN	logP(o/w)	TPSA Å²	% ABS
1	HPS/001	632.78	5	4	15	4.97	120.00	67.60
2	HPS/002	642.80	6	4	15	4.69	137.07	61.71
3	HPS/003	632.78	5	4	16	4.43	120.00	67.60
4	HPS/004	642.80	6	4	15	4.69	137.07	61.71
5	**Lopinavir**	628.814	5	4	15	5.19	120.00	67.60
6	HPS/005	720.98	3	5	19	4.58	129.99	64.15
7	HPS/006	698.93	3	6	18	3.09	139.22	60.97
8	HPS/007	700.95	3	6	18	3.06	139.22	60.97
9	HPS/008	676.91	3	5	18	4.67	125.55	65.69
10	**Ritonavir**	720.96	4	6	18	5.00	145.78	58.71
11	HPS/009	519.66	5	3	14	2.83	131.19	63.74
12	HPS/010	505.64	5	3	13	2.39	131.19	63.74
13	HPS/011	505.64	5	3	13	2.39	131.19	63.74
14	HPS/012	531.67	5	3	13	2.78	131.19	63.74
15	**Fosamprenavir**	583.60	5	2	13	1.40	171.40	49.87
16	HPS/013	489.64	4	2	13	3.40	110.96	70.72
17	HPS/014	489.64	4	2	13	3.40	110.96	70.72
18	HPS/015	462.59	3	2	13	3.07	124.79	65.95
19	HPS/016	490.62	5	2	13	3.06	105.17	72.72
20	**Amprenavir**	505.64	5	3	11	2.39	131.19	63.74
21	HPS/017	465.64	4	2	12	1.71	120.74	67.34
22	HPS/018	574.73	6	4	14	3.56	131.48	63.64
23	HPS/019	532.69	3	2	14	4.48	133.23	63.03
24	HPS/020	575.71	6	4	17	3.49	132.89	63.15
25	**Atazanavir**	704.87	7	5	14	4.74	171.22	49.93
26	HPS/021	573.71	6	3	13	2.70	126.43	65.38
27	HPS/022	595.72	6	3	13	3.37	140.42	60.56
28	HPS/023	574.72	6	2	14	2.84	117.41	68.49
29	HPS/024	546.66	5	2	12	2.49	145.46	58.81
30	**Darunavir**	547.70	3	9	12	2.9	149	51.41
31	HPS/025	645.89	7	3	14	5.01	125.04	65.86
32	HPS/026	661.89	8	4	14	4.86	145.27	59.92
33	HPS/027	661.89	8	4	14	4.86	145.27	59.92
34	HPS/028	569.81	6	5	12	5.35	105.06	72.75
35	**Nelfinavir**	547.673	6	3	11	2.436	140.42	60.56

MW = molecular weight; a_don = number of hydrogen bond donors; a_acc = number of hydrogen bond acceptors; b_1rotN = number of rotatable hydrogen bonds; logP(o/w) = octanol-water partition coefficient; TPSA = topological polar surface area.

**Table 2:** Binding free energy (kcal/mol) and the HIV protease inhibitory activity of selected hits from different reference drugs.

Ref Drug	S/N	Comp Code	ΔG (kcal/mol)	IC_50_ (µM)	% Inhibition
**Lopinavir**	1	HPS/001	−8.28	304.89	90.15
	2	HPS/002	−8.24	48.9	90.13
	3	HPS/003	−7.91	318.74	82.65
	4	HPS/004	−7.88	48.9	90.13
	5	**Lopinavir**	−7.80	203.69	90.12
**Ritonavir**	6	HPS/005	−8.98	7.79	59.07
	7	HPS/006	−8.63	31.17	59.8
	8	HPS/007	−8.57	36.33	60.78
	9	HPS/008	−8.54	14.58	59.48
	10	**Ritonavir**	−8.35	24.99	53.74
**Fosamprenavir**	11	HPS/009	−7.31	−14.7	65.58
	12	HPS/010	−7.15	6.80	65.59
	13	HPS/011	−6.97	6.80	65.59
	14	HPS/012	−6.91	−16.04	65.57
	15	**Fosamprenavir**	−6.88	39.79	65.65
**Amprenavir**	16	HPS/013	−6.96	0.14	54.98
	17	HPS/014	−6.85	0.14	54.98
	18	HPS/015	−6.76	134.14	70.70
	19	HPS/016	−6.72	66.22	66.11
	20	**Amprenavir**	−6.69	6.8	65.59
**Atazanavir**	21	HPS/017	−8.09	28.40	59.85
	22	HPS/018	−7.87	−18.12	52.71
	23	HPS/019	−7.68	168.45	54.00
	24	HPS/020	−7.64	−2.52	56.87
	25	**Atazanavir**	−7.63	7.88	52.48
**Darunavir**	26	HPS/021	−8.00	50.02	61.53
	27	HPS/022	−7.50	28.2	58.64
	28	HPS/023	−7.28	1.38	61.04
	29	HPS/024	−7.29	−18.51	58.39
	30	**Darunavir**	−7.01	−0.3	55.17
**Nelfinavir**	31	HPS/025	−7.94	−3.48	50.47
	32	HPS/026	−7.84	28.4	58.79
	33	HPS/027	−7.60	4.92	58.79
	34	HPS/028	−7.59	1.78	56.16
	35	**Nelfinavir**	−7.58	9.51	50.47

**Table 3:** Binding interactions of the hit compounds with 2Q5K.

S/N	Compound	Ligand	Receptor	Interaction	Distance (Å)	E (kcal/mol)
1	HPS/002	O 1	O GLY 27	H-donor	2.94	−1.9
		C 21	O GLY 27	H-donor	3.21	−0.2
		C 58	OD2 ASP 30	H-donor	3.60	−0.2
		O 6	N ASP 29	H-acceptor	2.94	−1.6
		6-ring	CA GLY 27	pi-H	4.28	−0.2
		6-ring	CG1 ILE 50	pi-H	4.24	−0.4
2	HPS/004	C 21	O GLY 27	H-donor	3.52	−0.2
		O 3	N ILE 50	H-acceptor	3.37	−0.8
		O 6	N ASP 29	H-acceptor	3.22	−2.4
		6-ring	CA ILE 47	pi-H	4.54	−0.2
		6-ring	N GLY 48	pi-H	3.85	−1.2
3	HPS/006	S 1	OG1 THR80	H-donor	3.73	−1.2
		N 10	O GLY 27	H-donor	3.69	−0.2
		C 29	OD1 ASP 25	H-donor	3.12	−2.6
		S 1	CD1 ILE 54	H-acceptor	4.11	−0.2
		O 4	CA GLY 49	H-acceptor	3.30	−0.6
4	HPS/007	N 8	O ILE 50	H-donor	2.83	−9.6
		C 37	O GLY 52	H-donor	3.13	−0.7
		C 68	OG1 THR80	H-donor	3.44	−0.2
		C 75	OD2 ASP 25	H-donor	3.32	−0.3
		S 2	CD1 ILE 50	H-acceptor	4.27	−0.2
		6-ring	CD1 LEU 23	pi-H	4.41	−0.2
		6-ring	CD2 LEU 23	pi-H	3.88	−0.3
		5-ring	CD1 LEU 47	pi-H	3.72	−0.4
5	HPS/008	S 2	OD2 ASP 29	H-donor	3.54	−3.2
		S 2	OD1 ASP 30	H-donor	3.47	−0.2
		N 7	O GLY 27	H-donor	3.41	−0.6
		N 9	O GLY 48	H-donor	3.13	−0.6
		C 33	O GLY 27	H-donor	3.36	−0.2
		N 15	NZ LYS 45	H-acceptor	3.30	−2.8
		6-ring	CD1 ILE 47	pi-H	4.18	−0.8
		6-ring	N ILE 50	pi-H	3.92	−1.0
6	HPS/009	C 36	O GLY 27	H-donor	3.61	−0.3
		O 7	N ASP 29	H-acceptor	3.05	−1.9
		6-ring	CA GLY 27	pi-H	3.63	−0.6

**Table 4:** Absorption and distribution (pkCSM).

		Absorption							Distribution			
S/N	Comp Code	Water Solubility	Caco2 Perm	Intestine Abs	Skin Perm.	P-GPS	P-GP1I	P-GP2I	VDss	FU	BBB Perm.	CNS Perm.
1	HPS/001	−4.788	−0.162	60.891	−2.735	Yes	Yes	Yes	−0.362	0	−1.011	−3.069
2	HPS/002	−4.625	0.271	61.515	−2.735	Yes	Yes	Yes	−0.399	−0.399	−0.712	−3.11
3	HPS/003	−4.741	−0.246	71.033	−2.735	Yes	Yes	Yes	−0.31	0.019	−1.35	−3.154
4	HPS/004	−4.625	0.271	61.515	61.515	Yes	Yes	Yes	−0.399	0.012	−0.712	−3.11
5	**Lopinavir**	−2.892	1.497	76.395	−2.735	No	No	No	0.011	0.381	−1.525	−1.418
6	HPS/005	−3.84	0.216	70.919	−2.735	Yes	Yes	Yes	0.702	0	−1.76	−3.362
7	HPS/006	−4.11	0.737	71.415	−2.735	Yes	Yes	Yes	0.302	0.048	−1.844	−3.642
8	HPS/007	−3.209	0.464	69.849	−2.735	Yes	Yes	Yes	1.105	1.105	−1.789	−3.895
9	HPS/008	−4.232	0.561	79.662	−2.735	Yes	Yes	Yes	0.273	0	−1.678	−3.23
10	**Ritonavir**	−3.358	0.377	69.45	−2.735	Yes	Yes	Yes	0.429	0	−1.665	−3.295
11	HPS/009	−3.315	0.356	66.625	−2.741	Yes	Yes	No	0.639	0.093	−1.081	−3.461
12	HPS/010	−3.312	0.354	65.884	−2.742	Yes	Yes	No	0.552	0.079	−1.018	−3.473
13	HPS/011	−3.312	0.354	65.884	−2.742	Yes	Yes	No	0.552	0.079	−1.018	−3.473
14	HPS/012	−3.499	0.484	71.755	−2.74	Yes	Yes	No	0.603	0.03	−0.974	−3.413
15	**Fosamprenavir**	−3.239	0.201	76.433	−2.735	Yes	Yes	No	0.228	0.12	−1.816	−4.025
16	HPS/013	−3.631	0.467	81.852	−2.746	Yes	Yes	No	0.569	0.012	−0.919	−2.991
17	HPS/014	−3.631	0.467	81.852	−2.746	Yes	Yes	No	0.569	0.012	−0.919	−2.991
18	HPS/015	−3.494	−0.099	65.218	−2.735	Yes	No	No	−0.556	0.089	−1.022	−3.197
19	HPS/016	−4.52	0.613	73.02	−2.812	Yes	Yes	No	0.127	0	−1.033	−3.333
20	**Amprenavir**	−3.312	0.354	65.884	−2.742	Yes	Yes	No	0.552	0.079	−1.018	−3.473
21	HPS/017	−3.028	0.394	74.785	−2.755	Yes	Yes	Yes	1.614	0.361	−0.792	−2.976
22	HPS/018	−3.511	−0.247	63.672	−2.735	Yes	Yes	Yes	0.325	0.107	−1.222	−3.158
23	HPS/019	−3.278	−0.116	46.777	−2.735	Yes	No	Yes	0.143	0.222	−0.925	−2.715
24	HPS/020	−4.316	0.288	60.286	−2.741	Yes	Yes	Yes	0.053	0.05	−0.964	−3.071
25	**Atazanavir**	−3.849	0.294	45.728	−2.735	Yes	Yes	Yes	0.143	0.03	−1.352	−3.224
26	HPS/021	−3.381	0.457	80.889	−2.725	Yes	Yes	No	0.776	0.046	−1.076	−3.474
27	HPS/022	−3.796	0.358	82.453	−2.735	Yes	Yes	Yes	0.208	0	−1.115	−3.273
28	HPS/023	−3.401	0.51	79.453	−2.738	Yes	Yes	Yes	0.148	0.242	−1.15	−3.47
29	HPS/024	−3.289	−3.289	49.063	−2.735	Yes	No	No	0.06	0.164	−0.921	−3.656
30	**Darunavir**	−3.348	0.493	75.477	−2.739	Yes	Yes	No	−2.739	0.055	−1.111	−3.519
31	HPS/025	−4.568	0.777	69.402	−2.73	Yes	Yes	Yes	0.129	0.052	−0.935	−2.849
32	HPS/026	−3.711	0.265	66.903	−2.735	Yes	Yes	Yes	0.31	0.139	−1.099	−2.911
33	HPS/027	−3.711	0.265	66.903	−2.735	Yes	Yes	Yes	0.31	0.139	−1.099	−2.911
34	HPS/028	−3.107	0.575	70.605	−2.735	Yes	Yes	Yes	0.831	0.294	−0.77	−2.498
35	**Nelfinavir**	−3.894	0.693	70.888	−2.737	Yes	Yes	Yes	0.563	0.094	−0.522	−2.245

Caco2 perm.—Caco2 permeability (log Papp in 10^−6^ cm/s); IA(H)—Intestinal absorption (human); Skin perm—Skin Permeability (log Kp); P-GPS—P-glycoprotein substrate; P-GP1I—P-glycoprotein I inhibitor; P-GP2I—P-glycoprotein II inhibitor; VDss—Volume of distribution at steady state (human) (Log L/kg); FU—Fraction unbound (human) (Fu); BBB perm.—BBB permeability (log BB); CNS perm.—CNS permeability (log PS).

**Table 5:** Metabolism and excretion (pkCSM).

		Metabolism							Excretion	
S/N	Comp Code	CYP2D6 Substrate	CYP3A4 Substrate	CYP1A2 Inhibitor	CYP2C19 Inhibitor	CYP2C9 Inhibitor	CYP2D6 Inhibitor	CYP3A4 Inhibitor	Total Clearance (log ml/min/kg)	Renal OCT2 Substrate
1	HPS/001	No	Yes	No	Yes	No	No	Yes	0.335	No
2	HPS/002	No	Yes	No	No	No	No	Yes	0.429	Yes
3	HPS/003	No	Yes	No	Yes	Yes	No	Yes	0.371	No
4	HPS/004	No	Yes	No	No	No	No	Yes	0.429	No
5	**Lopinavir**	No	No	Yes	No	No	No	No	−114.036	No
6	HPS/005	No	Yes	No	No	Yes	No	Yes	0.613	No
7	HPS/006	No	Yes	No	No	No	No	Yes	1.122	No
8	HPS/007	No	Yes	No	No	No	No	Yes	1.202	No
9	HPS/008	No	Yes	No	Yes	Yes	No	Yes	0.374	No
10	**Ritonavir**	No	Yes	No	No	Yes	No	Yes	0.564	No
11	HPS/009	No	Yes	No	No	No	No	Yes	1.021	No
12	HPS/010	No	Yes	No	No	No	No	Yes	0.918	No
13	HPS/011	No	Yes	No	No	No	No	Yes	0.918	No
14	HPS/012	No	Yes	No	No	No	No	Yes	0.755	No
15	**Fosamprenavir**	No	Yes	No	No	No	No	Yes	0.282	No
16	HPS/013	No	Yes	No	Yes	Yes	No	Yes	0.782	No
17	HPS/014	No	Yes	No	Yes	Yes	No	Yes	0.782	No
18	HPS/015	No	No	No	No	No	No	No	1.06	No
19	HPS/016	No	Yes	No	Yes	No	No	Yes	1.13	No
20	**Amprenavir**	No	Yes	No	No	No	No	Yes	0.918	No
21	HPS/017	No	Yes	No	No	No	Yes	Yes	0.793	No
22	HPS/018	No	Yes	No	Yes	Yes	No	Yes	0.375	No
23	HPS/019	No	Yes	No	No	No	No	No	0.378	No
24	HPS/020	No	Yes	No	Yes	Yes	No	Yes	0.767	No
25	**Atazanavir**	No	Yes	No	No	Yes	No	Yes	0.258	No
26	HPS/021	No	Yes	No	No	No	No	Yes	0.439	No
27	HPS/022	No	Yes	No	No	Yes	No	Yes	0.467	No
28	HPS/023	No	Yes	No	No	No	No	Yes	0.491	No
29	HPS/024	No	Yes	No	No	No	No	No	0.654	No
30	**Darunavir**	No	Yes	No	No	No	No	Yes	0.622	No
31	HPS/025	No	Yes	No	No	No	No	Yes	0.439	No
32	HPS/026	No	Yes	No	No	No	No	Yes	−0.117	No
33	HPS/027	No	Yes	No	No	No	No	Yes	−0.117	No
34	HPS/028	Yes	Yes	No	No	No	Yes	No	0.447	No
35	**Nelfinavir**	No	Yes	No	Yes	No	Yes	Yes	0.399	No

**Table 6:** The results of the toxicity test with ProToxII.

S/N	Comp Code	LD_50_ mg/kg	Toxici-ty Class	Average Similarity	Prediction Accuracy %	Hepatotoxicity	Carcino-Genicity	Immuno-Toxicity	Muta-Genicity	Cytotoxicity	Aroma-Tase	Estrogen Receptor-α	Androgen Receptor	PPAR-γ
1	HPS/001	5000	V	61.43	68.07	Inactive ^a^	Inactive ^a^	Inactive ^a^	Inactive ^a^	Inactive ^a^	Inactive ^a^	Inactive ^a^	Inactive ^a^	Inactive ^a^
2	HPS/002	5000	V	58.87	67.38	Inactive ^a^	Inactive ^a^	Inactive ^a^	Inactive ^a^	Inactive ^a^	Inactive ^a^	Inactive ^a^	Inactive ^a^	Inactive ^a^
3	HPS/003	5000	V	61.99	68.07	Inactive ^a^	Inactive	Inactive ^a^	Inactive ^a^	Inactive ^a^	Inactive ^a^	Inactive ^a^	Inactive ^a^	Inactive ^a^
4	HPS/004	5000	V	58.87	67.38	Inactive ^a^	Inactive ^a^	Inactive ^a^	Inactive ^a^	Inactive ^a^	Inactive ^a^	Inactive ^a^	Inactive ^a^	Inactive ^a^
5	**Lopinavir**	5000	V	59.88	67.38	Inactive ^a^	Inactive ^a^	Inactive ^a^	Inactive ^a^	Inactive ^a^	Inactive ^a^	Inactive ^a^	Inactive ^a^	Inactive ^a^
6	HPS/005	1000	IV	40.75	54.26	Active	Inactive	Inactive	Inactive	Inactive	Inactive ^a^	Inactive ^a^	Inactive ^a^	Inactive ^a^
7	HPS/006	800	IV	42.36	54.26	Inactive	Inactive	Inactive ^a^	Inactive	Inactive	Inactive ^a^	Inactive ^a^	Inactive ^a^	Inactive ^a^
8	HPS/007	1000	IV	42.53	54.26	Inactive	Inactive	Inactive ^a^	Inactive	Inactive	Inactive ^a^	Inactive ^a^	Inactive ^a^	Inactive ^a^
9	HPS/008	500	IV	40.28	54.26	Inactive	Inactive	Inactive ^a^	Inactive	Inactive	Inactive ^a^	Inactive ^a^	Inactive ^a^	Inactive ^a^
10	**Ritonavir**	1000	IV	42.32	54.26	Active ^a^	Inactive	Inactive ^a^	Inactive ^a^	Inactive	Inactive ^a^	Inactive ^a^	Inactive ^a^	Inactive ^a^
11	HPS/009	300	III	48.58	54.26	Inactive	Inactive	Inactive ^a^	Inactive ^a^	Inactive	Inactive ^a^	Inactive ^a^	Inactive ^a^	Inactive ^a^
12	HPS/010	300	III	48.58	54.26	Inactive ^a^	Inactive	Inactive ^a^	Inactive ^a^	Inactive	Inactive ^a^	Inactive ^a^	Inactive ^a^	Inactive ^a^
13	HPS/011	300	III	48.58	54.26	Inactive ^a^	Inactive	Inactive ^a^	Inactive ^a^	Inactive	Inactive ^a^	Inactive ^a^	Inactive ^a^	Inactive ^a^
14	HPS/012	300	III	48.12	54.26	Inactive	Inactive	Inactive ^a^	Inactive ^a^	Inactive	Inactive ^a^	Inactive ^a^	Inactive ^a^	Inactive ^a^
15	**Fosamprenavir**	300	III	43.99	54.26	Inactive ^a^	Inactive	Inactive ^a^	Inactive	Inactive	Inactive ^a^	Inactive ^a^	Inactive ^a^	Inactive ^a^
16	HPS/013	300	III	50.25	67.38	Inactive ^a^	Inactive	Inactive ^a^	Inactive ^a^	Inactive	Inactive ^a^	Inactive ^a^	Inactive ^a^	Inactive ^a^
17	HPS/014	300	III	50.25	67.38	Inactive ^a^	Inactive	Inactive ^a^	Inactive ^a^	Inactive	Inactive ^a^	Inactive ^a^	Inactive ^a^	Inactive ^a^
18	HPS/015	2500	V	51.48	67.38	Inactive	Inactive	Inactive ^a^	Inactive ^a^	Inactive	Inactive ^a^	Inactive ^a^	Inactive ^a^	Inactive ^a^
19	HPS/016	1000	IV	48.28	54.26	Inactive ^a^	Inactive	Inactive ^a^	Inactive ^a^	Inactive	Inactive ^a^	Inactive ^a^	Inactive ^a^	Inactive ^a^
20	**Amprenavir**	300	III	48.58	54.26	Inactive ^a^	Inactive	Inactive ^a^	Inactive ^a^	Inactive	Inactive ^a^	Inactive ^a^	Inactive ^a^	Inactive ^a^
21	HPS/017	200	III	47.45	54.26	Active	Active	Active ^a^	Inactive	Inactive	Inactive ^a^	Inactive ^a^	Inactive ^a^	Inactive ^a^
22	HPS/018	200	III	44.60	54.26	Inactive	Inactive	Inactive ^a^	Inactive	Inactive	Inactive ^a^	Inactive ^a^	Inactive ^a^	Inactive ^a^
23	HPS/019	500	IV	60.36	68.07	Inactive	Inactive	Inactive ^a^	Inactive ^a^	Inactive	Inactive ^a^	Inactive ^a^	Inactive ^a^	Inactive ^a^
24	HPS/020	200	III	48.94	54.26	Inactive	Inactive	Inactive ^a^	Inactive	Inactive	Inactive ^a^	Inactive ^a^	Inactive ^a^	Inactive ^a^
25	**Atazanavir**	200	III	47.65	54.26	Active	Inactive	Inactive ^a^	Inactive	Inactive	Inactive ^a^	Inactive ^a^	Inactive ^a^	Inactive ^a^
26	HPS/021	245	III	48.68	54.26	Active	Inactive	Active	Inactive	Inactive	Inactive ^a^	Inactive ^a^	Inactive ^a^	Inactive ^a^
27	HPS/022	245	III	48	54.26	Active	Inactive	Active	Inactive	Inactive	Inactive ^a^	Inactive ^a^	Inactive ^a^	Inactive ^a^
28	HPS/023	245	III	50.43	67.38	Active	Active	Active	Inactive	Inactive	Inactive ^a^	Inactive ^a^	Inactive ^a^	Inactive ^a^
29	HPS/024	3000	V	47.04	54.26	Active	Inactive	Inactive ^a^	Inactive	Inactive	Inactive ^a^	Inactive ^a^	Inactive ^a^	Inactive ^a^
30	**Darunavir**	245	III	52.42	67.38	Active ^a^	Inactive	Inactive	Inactive	Inactive	Inactive ^a^	Inactive ^a^	Inactive ^a^	Inactive ^a^
31	HPS/025	1000	IV	45.27	54.26	Inactive	Inactive	Active ^a^	Inactive	Inactive	Inactive ^a^	Inactive ^a^	Inactive ^a^	Inactive ^a^
32	HPS/026	1000	IV	43.19	54.26	Inactive	Inactive	Active ^a^	Inactive	Inactive	Inactive ^a^	Inactive ^a^	Inactive ^a^	Inactive ^a^
33	HPS/027	1000	IV	43.19	54.26	Inactive	Inactive	Active ^a^	Inactive	Inactive	Inactive ^a^	Inactive ^a^	Inactive ^a^	Inactive ^a^
34	HPS/028	1000	IV	45.52	54.26	Inactive	Inactive	Active ^a^	Inactive ^a^	Inactive	Inactive ^a^	Inactive ^a^	Inactive ^a^	Inactive ^a^
35	**Nelfinavir**	600	IV	49.30	54.26	Inactive	Inactive	Active ^a^	Inactive ^a^	Inactive	Inactive ^a^	Inactive ^a^	Inactive ^a^	Inactive ^a^

^a^ High probability (≥0.70); Peroxisome Proliferator Activated Receptor Gamma (PPAR-Gamma).

**Table 7:** The results of the toxicity evaluation with pkCSM.

S/N	Comp Code	AMES Toxicity	Max. Tolerated Dose (Human)	hERG I Inhibitor	hERG II Inhibitor	(LD_50_) mol/kg	(LOAEL) log mg/kg_bw/day	Hepatotoxicity	Skin Sensitization	T.Pyriformis Toxicity	Minnow Toxicity
1	HPS/001	No	−0.272	No	Yes	2.413	3.504	Yes	No	0.286	−1.564
2	HPS/002	No	−0.254	No	Yes	2.38	2.979	Yes	No	0.285	−1.219
3	HPS/003	No	−0.198	No	Yes	2.8	3.389	Yes	No	0.285	−2.213
4	HPS/004	No	−0.254	No	Yes	2.674	3.842	Yes	No	0.285	−1.219
5	**Lopinavir**	No	−0.297	No	Yes	2.382	5.949	Yes	No	0.286	−1.501
6	HPS/005	No	−0.22	No	Yes	2.818	3.631	Yes	No	0.285	4.55
7	HPS/006	No	−0.224	No	Yes	3.206	2.454	Yes	No	0.285	1.04
8	HPS/007	No	−0.3	No	Yes	2.714	2.126	Yes	No	0.285	1.197
9	HPS/008	No	−0.308	No	Yes	2.661	2.169	Yes	No	0.286	1.535
10	**Ritonavir**	No	0.096	No	Yes	2.703	2.231	Yes	No	0.285	1.787
11	HPS/009	No	0.021	No	No	2.578	1.744	Yes	No	0.285	−3.865
12	HPS/010	No	0	No	No	2.617	1.767	Yes	No	0.285	−4.033
13	HPS/011	No	0	No	No	2.617	1.767	Yes	No	0.285	−4.033
14	HPS/012	No	−0.16	No	No	2.69	1.663	Yes	No	0.285	−4.327
15	**Fosamprenavir**	No	−0.029	No	No	2.396	2.151	Yes	No	0.285	−4.393
16	HPS/013	No	−0.355	No	No	2.415	1.989	Yes	No	0.293	−3.9
17	HPS/014	No	−0.355	No	No	2.415	1.989	Yes	No	0.293	−3.9
18	HPS/015	No	−0.29	No	No	2.282	1.904	Yes	No	0.285	−1.634
19	HPS/016	No	−0.048	No	No	3.255	1.823	Yes	No	0.286	−4.231
20	**Amprenavir**	No	0	No	No	2.617	1.767	Yes	No	0.285	−4.033
21	HPS/017	Yes	−0.295	No	Yes	2.528	1.731	Yes	No	0.286	0.934
22	HPS/018	No	0.178	No	Yes	2.805	5.592	Yes	No	0.285	1.481
23	HPS/019	No	0.256	No	Yes	2.567	4.603	Yes	No	0.285	0.939
24	HPS/020	No	−0.419	No	Yes	3.317	0.624	Yes	No	0.286	0.553
25	**Atazanavir**	No	−0.16	No	No	2.665	2.703	Yes	No	0.285	2.075
26	HPS/021	No	−0.914	No	No	2.702	1.716	Yes	No	0.29	0.501
27	HPS/022	No	−0.353	No	Yes	2.434	2.96	Yes	No	0.286	−0.052
28	HPS/023	No	−0.504	No	Yes	2.088	2.666	Yes	No	0.285	0.463
29	HPS/024	No	−0.105	No	No	2.535	2.336	Yes	No	0.285	1.082
30	**Darunavir**	No	−0.763	No	No	2.107	2.775	Yes	No	0.289	0.61
31	HPS/025	No	−0.831	No	Yes	2.566	2.949	Yes	No	0.29	0.501
32	HPS/026	No	−0.575	No	Yes	2.538	3.508	Yes	No	0.285	1.725
33	HPS/027	No	−0.575	No	Yes	2.538	3.508	Yes	No	0.285	1.725
34	HPS/028	No	0.019	No	Yes	2.379	4.271	Yes	No	0.285	2.047
35	**Nelfinavir**	No	−0.576	No	Yes	2.54	3.911	Yes	No	0.287	1.236

## Data Availability

Not applicable.

## Supplementary Materials

The following supporting information can be downloaded at: https://www.mdpi.com/article/10.3390/ijms232012149/s1.
