# Supplementary material for: Discovery of Novel HIV Protease Inhibitors Using Modern Computational Techniques
Source: Int J Mol Sci. 2022 Oct 12;23(20):12149. doi: 10.3390/ijms232012149 (PMC9603388; doi:10.3390/ijms232012149)
Supplement: Supplementary file 1 [file ijms-23-12149-s001.zip › ijms-1907053-supplementary.pdf]

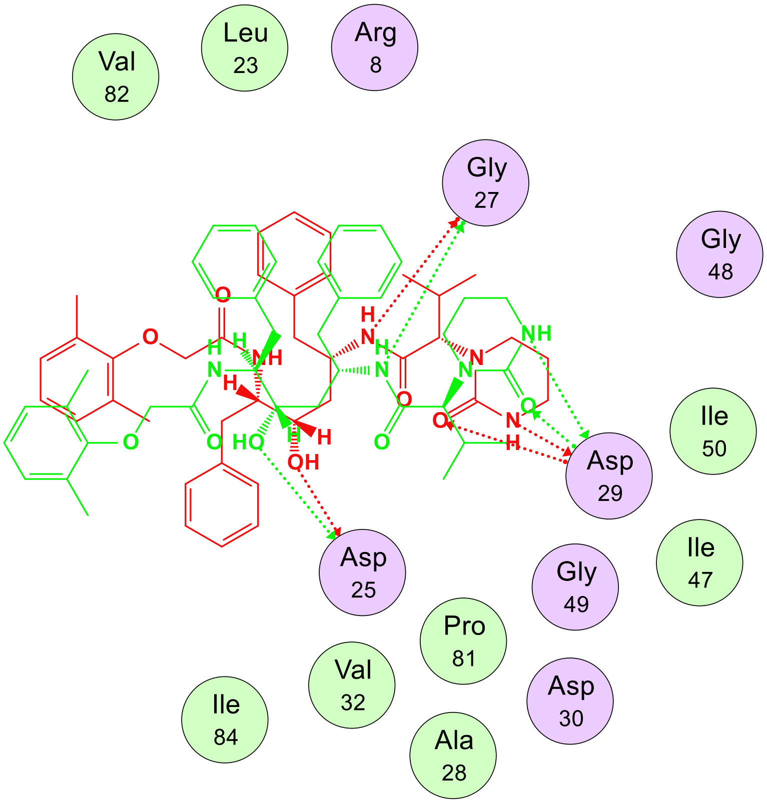

Figure S1: The 2D representation of the docked lopinavir (red) overlaid on the co-crystallized lopinavir (green) in the active binding site of 2Q5K

This again, elucidates the validity of the docking protocol. Both the docked and the co-crystallized lopinavir shared the same amino acid residues (ASP 25, GLY 27 and ASP 29) which are the catalytic triads

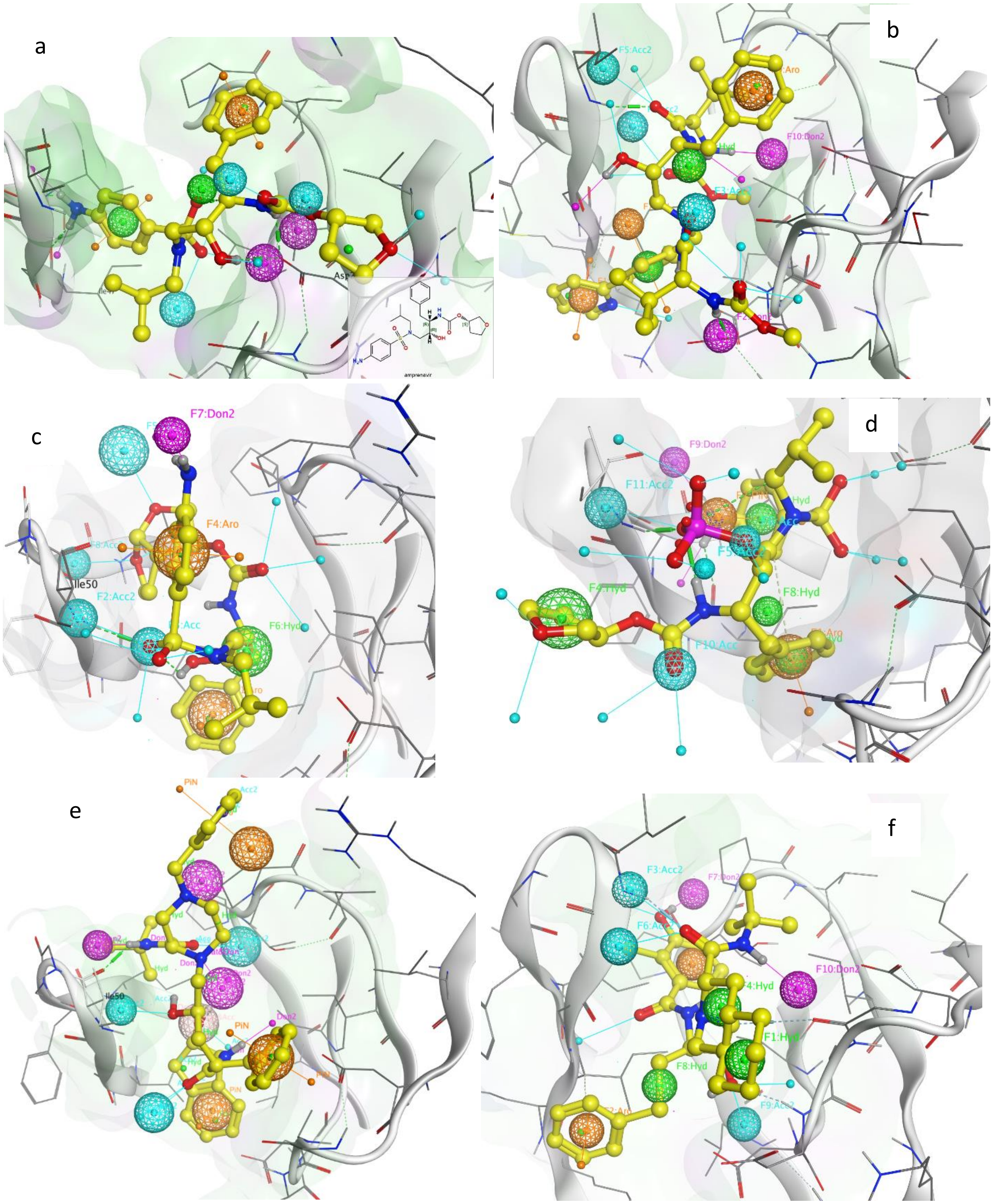

Figure S2: Pharmacophore annotations of (a) Amprenavir (b) atazanavir (c) darunavir (d) fosamprenavir (e) Indinavir (f) Nelfinavir

Key: H-bond donor – Don (pink), H-bond acceptor – Acc (cyan), hydrophobic Atom – HydA (green); projected donor – Don2 (pink), projected acceptor – Acc2 (cyan), aromatic – Aro (orange)
